# An Account of Two Cases of Violent Constipation of the Bowels; the First Successfully Treated by the Internal and External Application of Cold Water; and the Second Terminating by a Discharge of Matter from the Vagina

**Published:** 1787

**Authors:** Charles Kite

**Affiliations:** Member of the Corporation of Surgeons of London, and Surgeon at Gravesend, in Kent


XI. *An Account of two Cases of violent Constipation of the Bowels; the first successfully treated by the internal and external Application of cold Water; and the second terminating by a Discharge of Matter from the Vagina. Communicated in a Letter to Dr.* Simmons, *F. R. S. by Mr.* Charles Kite, *Member of the Corporation of Surgeons of London, and Surgeon at Gravesend, in Kent.*

I TAKE the liberty, Sir, of submitting the two following cases to your consideration. The termination of the first is decisively and unequivocally in favour of a plan, which at present does not seem sufficiently regarded; and as it tends in a remarkable manner to confirm the propriety of a practice which is but imperfectly understood, I hope it may may prove one means of introducing it more generally into practice.

The cure of the second case is entirely unconnected with the first; yet, as they resemble each other in their nature, as it originated from a cause, which, so far as I recollect, is not mentioned by any writer, and as the causes of the generality of these cases are involved in much obscurity, I presume an account of it may prove not altogether unacceptable to you.

### CASE I.

Daniel Donaldson, of a strong, robust constitution, forty-eight years old, and formerly a sailor, till October, 1785, enjoyed a good state of health; but at that time, while he resided in a workhouse in some part of Lincolnshire, was seized with an irregular intermittent. It continued about three months, and then, by taking a very few medicines, (among which he does not believe there was any bark) it left him. From this period he dated the origin of his complaint; for soon after he was subject to pains in various parts of the abdomen, but more especially in the left hypochondrium and round the navel. When the pains were violent, the part affected became swelled, and the bowels were coſtive; but on ſtools being procured, he immediately grew eaſy, and the ſwelling diſappeared. He ſoon perceived that when a ſufficiently large quantity of fæces was accumulated, the ſame ſymptoms returned, and he was obliged to have recourſe to ſalts, or ſome other purging medicine, in order to obtain ſtools. In this manner was he generally attacked once every four or five days; but as the remedies he commonly made uſe of had always given a temporary relief, I was not deſired to ſee him till the 23d of March, 1786, when he was taken much worſe, in conſequence of the medicine having failed in producing its uſual effect.

The pain, with ſome degree of tenſion, was general all over the abdomen, but immediately below the navel it was more ſevere. At this part there was a conſiderable ſwelling, which, at firſt, ſeemed a contraction of the abdominal muſcles, but afterwards it appeared more likely to be a collection of air or fæces confined in ſome part of the bowels. He had paſſed no ſtools for about a week, and his urine had been made frequently, and in ſmall quantity; but there was no appearance of inflammation or fever, for no rigours had attended: the pulſe was was ſcarcely altered from a healthy ſtate, and as yet he was not attacked either with ſickneſs or vomiting.

Previous to my ſeeing him he had taken three ounces of ſalts, which had produced no effect; a ſtrong doſe of jalap and cream of tartar was then given, with no better ſucceſs. Extract. cathart. and calomel having given relief in a former fit, were now exhibited in large quantities, but with no advantage; at the ſame time clyſters were had recourſe to, which ſometimes were retained, but frequently voided in the ſame ſtate as when injected. It ſeems unneceſſary to ſpecify to you every particular remedy which was made uſe of. It will ſuffice, perhaps, to mention, that, after bleeding, the purging ſalts, infuſion of ſenna, jalap, extract. cathart. calomel, caſtor oil, &c. were by turns employed; and as they occaſioned neither ſickneſs nor increaſe of pain, they were all given in much larger doſes than I had ever ventured on myſelf, or than I had known given by others. The clyſters were emollient, oily, and purgative; ſometimes they were formed with a ſolution of turpentine, and frequently with a ſtrong infuſion of tobacco: the ufual quantity of each clyfter was a pint; this was ordered to be forcibly injected through a pipe with a bore larger than ufual.

The ftate of the inferior part of the rectum had previoufly been afcertained, but I now thought it advifeable to examine whether any conftriction exifted in the lower part of the colon. With this intention, a candle, nearly a foot in length, was carefully introduced, but not the leaft obftruction was perceived: it was, however, fuffered to remain till the tallow melted; and conceiving fome benefit might arife from a foft fubftance lying fome time in the part, this remedy was again repeated.

The warm bath was now ufed; but it was evident he was in much greater pain while in it than before. As foon as he came out, a clyfter of the fumes of tobacco was blown up the rectum: he was again put into the bath, and while in it another fmoke clyfter was injected, and one more was repeated when he came from it.

The fame day a fmall quantity of cold water was fprinkled on his legs and arms, while he lay on a blanket in a warm room; but the next he was fupported on the cold ftones of a wafhhoufe houfe perfectly naked, and this during a fevere froft, while a pailful of cold water was, at different times, dafhed over his legs and thighs and poured down his arms. This, inftead of increafing the pain, as the warm bath had done, made him much eafier: the relief, how ever, was but of fhort continuance; but it was the only effect it produced.

The day following, after a tobacco-fmoke clyfter had been given, he was fick, and vomited much. What he brought up tafted powerfully of the tobacco, and bore an exact refemblance, both in appearance and fmell, to the liquid fæces which were forced from him by the violent effort of ftraining. Trivial as this evacuation was, yet, when the ficknefs had fubfided, he thought himfelf eafier for it; I therefore encouraged the vomiting, by giving half a fcruple of vitriol. alb. every half hour till it operated, which it foon did, once or twice, and with fimilar effect.

Every meafure had now been employed, from which I could fuggeft the moft diftant probability of fuccefs; and the writings of the moft eminent among the ancient as well as modern practitioners were in vain ranfacked for

 new new remedies *. To thofe which I had ufed a fair and unprejudiced trial had been given. In particular, a liberal and almoft unreftrained ufe had been made of the ftrongeft purgatives, opium, æther, injections of every kind, (amounting, in number, altogether to fifty) electricity, the warm bath, the application of cold water, remedies fo juftly extolled, and fo much relied on in the advanced ftages of thefe complaints, but without the leaft fuccefs.

When I faw him on the fifteenth day of the difeafe, I found him in the following ftate: — The bowels continued obftinately conftipated; the belly was hard, and immediately below the navel it was fwelled fomewhat irregularly: the pain was violent, but tenfive, at times remitting, and increafing much on preffure. The vomitings were frequent, fometimes of a flimy matter, at others ftercoraceous, having both the fmell and appearance of liquid ftools †. The pulfe was foft, weak, and irregular; the

* I muft except quickfilver, againft which the concurrent teftimony of many refpectable authorities, as well as common fenfe, militate fo powerfully, that I did not ufe it.

† Once being fick after a tobacco-fmoke clyfter, what was brought up, he faid, tafted ftrongly of tobacco; but I could never learn that any other clyfter had a fimilar effect.

tongue brown, but moiſt; the eyes ſunk in the ſockets, dull and heavy; the breathing ſhort, frequent, and attended with conſtant motion of the noſtrils; the hiccup was frequent and harraſſing; his appetite and ſleep had almoſt forſaken him; he had often a ſubſultus, ſometimes a tendency to delirium, and his urine was ſcantily ſecreted, and frequently voided with ſome pain, depoſiting a copious brown ſediment on ſtanding.

The patient had hitherto ſuſtained his complaint with great fortitude and reſolution, and had ſuffered every plan to be put in execution with ſingular patience; but being now become ſenſible of his extreme danger, he was anxious and dejected; deſpair was ſettled in his countenance, and he requeſted he might be permitted to die peaceably. This was his ſituation, and ſo dreadful did it appear, that an alteration for the better ſcarce entered my mind.

I join thoſe in opinion who think it better, in deſperate caſes, to have recourſe to doubtful or even dangerous remedies, than ſuffer the patient to be loſt without making uſe of any means to ſave him. Were we to obſerve this as an invariable rule; were we never to relinquiſh our attempts till they can no longer be employed, it would, I am confident, be productive of many extraordinary recoveries. Every practitioner, who is guided by these sentiments, can, doubtlefs, bring to mind feveral inftances wherein his apparently vain and fruitlefs perfeverance has been crowned with the moft unexpected fuccefs. The termination, however, of the prefent cafe is fo decifively in point, that it is unneceffary to adduce any farther proof in fupport of the opinion. Actuated by this principle, and revolving in my mind the effect of the various articles which had been ufed, I could but obferve, that, although no evacuation followed the application of the cold water, yet the patient was evidently eafier after it; whereas quite the reverfe was the cafe while he was in the warm bath; for he was then in greater pain than ufual. This determined me once more to make trial of that remedy; but, in order to derive any material advantage from it, I was perfuaded it would be neceffary to urge it to a much greater length than I had hitherto. This was accordingly done, and to fuch a degree, that nothing but the extreme danger of the patient could juftify my having recourfe to fuch defperate proceeding.

As he was now much too weak to be removed into the waſhhouſe, he was ſupported, ſitting on the ſide of the bed, with his feet in a tub. In this ſituation two or three pails full of the coldeſt water were poured over his legs and thighs, ſo that his feet and ancles were of courſe conſtantly immerſed in the liquid. This operation was perpetually repeated for the ſpace of ten minutes, when he was ſo much affected by the intenſe cold, that I judged it prudent to deſiſt. He was wiped dry, and put to bed. Within the half hour, being then pretty well recovered, a pint and a half of cold water was injected by clyſter, and almoſt immediately after wet napkins were applied cold to the whole abdomen, and renewed as ſoon as they became in the leaſt warm. The effect of this treatment was ſo ſtrongly marked, that it was really aſtoniſhing; for in a few minutes he had a profuſe evacuation of uncommonly hard and large fæces, and this was followed by ſeveral thinner ones. He was now comparatively eaſy: the ſwelling and hardneſs of the belly was conſiderably abated; he had no farther return of the vomiting or hiccup; and there was every appearance of ſpeedy recovery. The ſtools, however, notwithſtanding they were paſſed in great abundance, abundance, did not ſeem ſufficient to anſwer the intention completely. Several doſes of ſenna and ſalts, with warm relaxing and purging clyſters, were then again applied, but with no better ſucceſs than they had been before. At the expiration of two days I was apprehenſive our affairs were getting into the ſame channel as uſual, in conſequence of which I ordered the cold-water clyſters and wet cloths to be repeated, and allowed him to take half a pint of cold water every hour till he had taken a quart. Theſe again procured a tolerably good ſtool, and I was in hopes that a proper repetition of purging medicines by the mouth and rectum would now be able to effect their purpoſe. Still, however, I was miſtaken, and at the end of three days more I had the mortification to find his bowels were as obſtinately conſtipated as at firſt.

Upon attentively conſidering the caſe at this period, it occurred to me, that the firſt time the frigid operations were both internally and externally employed, the patient was extremely affected by the cold, and then a profuſe evacuation ſoon took place; but the ſecond time he ſeemed little affected by it, the evacuation was leſs, and it was longer before it was procured.

This

This determined me to proceed exactly in the same manner as I had done at firft. Accordingly, two pailfuls of water were poured over his legs and thighs till fuch time as he became extremely cold, and then the cold clyfters and cold cloths were applied. The event now fully anfwered my moft fanguine wifhes, for a profufe evacuation enfued, and I had the pleafure the next morning to find a common purgative had operated freely, and that the inteftines were now completely unloaded.

It may not be improper to obferve, that, notwithftanding the enormous and uncommonly large quantity of purging medicines which he had taken, fo far was a purging from enfuing, that it was neceffary to continue their ufe, once in two or three days, for fome time after the obftruction was removed.

In about three weeks after the patient had overcome this complaint, he became afcitic. As foon as this was perceived, diuretics of various kinds were had recourfe to, particularly fquills and fox glove, but without effect. Cream of tartar, in the quantity of an ounce, was given every day; blood and mucus were evacuated by it, but no water. Upon the prefumption that the liver was concerned in the production production of the difeafe, large dofes of mercurial ointment, with camphor, were rubbed over the region of that vifcus; and calomel, to the amount of fix grains a day, was, for fome time, adminiftered; but, at the expiration of three weeks, the fwelling had increafed fo much, and was fo painful, that it was neceffary to draw off the water. Nineteen pints were evacuated; on fubmitting part of it to a degree of heat fufficient to coagulate the ferum, part of it only coagulated, and that in an imperfect degree. The fox glove was again had recourfe to; one grain of the powder was given twice a day for a fortnight: it then occafioned ficknefs and flight vomiting, but no increafe of urine. He was tapped feveral times; but that, and in fhort every other remedy, proved merely palliative: he ftruggled till the beginning of December, and then died.

I very much wifhed to examine the ftate of the abdominal vifcera after death; but feveral circumftances concurred in preventing me. I was the lefs anxious, however, about it, as in the fpace of near nine months he had no return of the ftoppage in the bowels, except fuch as readily gave way to a mild dofe of common phyfic.

### CASE II.

About the middle of June, 1784, I was desired to see Mrs. Neville at Ludderdown, a married woman, aged twenty-seven, and somewhat inclined to corpulency.

She had lately recovered from lying-in of her second child. Her first labour was so violent and sudden, it was accomplished without the assistance of a midwife. The placenta separated very properly; but on the third day after delivery she was attacked with shivering, which continued three or four hours, and was succeeded by profuse flooding, which, when it had weakened her very considerably, ceased spontaneously. The second was equally as sudden as the first, not allowing time for the surgeon to come to her assistance. She was, on the third day, attacked with shivering and flooding, which, as before, stopped when it had very much reduced her.

Exactly one month after her last labour she was seized with severe, shooting, darting pains in the lower part of the abdomen, immediately above the pubis, occupying a space about the size of the palm of the hand, always fixed to that part, and never in the least wandering

 from from it. She began at the ſame time to be affected with fever and coſtiveneſs.

Theſe complaints continually increaſing, I was, on the ſeventh day, deſired to viſit her, when I received the preceding account.

The integuments of the abdomen were not ſwelled, or ſore to the touch; but on preſſing hard above the pubis the pain was very ſevere. No ſtools had paſſed for a week, except now and then a ſmall quantity about the ſize of a nut, and as hard as a ſtone; but ſhe had a conſtant deſire, and every quarter of an hour a violent ſtraining came on, of about five minutes continuance, during which time her pain was almoſt intolerable: in the remiſſions, however, ſhe was pretty eaſy, and now and then enjoyed ſhort intervals of refreſhing ſleep.

Her pulſe was weak, ſmall, and ſomewhat hard; the heat of the body was conſiderable, the ſkin was dry, and the urine made frequently, in ſmall quantities, high coloured, and without ſediment. Her countenance was ſometimes fluſhed, and much dejected, having the reſemblance of one worn out by fatigue; but at the ſame time it had that peculiar appearance, which I find myſelf unable to deſcribe, but which perſuaded me the complaint had not made that impreſſion impreſſion on the conſtitution which, from the hiſtory of the caſe, I ſhould have expected. Her tongue was white, rough, and dry; and ſhe was very frequently drinking ſmall quantities of ſome weak liquid to abate her thirſt.

I thought it adviſeable to try the effect of bleeding her; but when about eight ounces of blood were taken away, the pulſe lowered, and I immediately ſtopped: on ſtanding, the blood was ſomewhat buffy, but in other reſpects looked not amiſs.

Pills, compoſed of the cathartic extract, calomel, and opium, (a medicine I have often employed with ſingular advantage in ſevere coſtiveneſs) were recommended to be taken every half hour till they produced the deſired effect. Purging clyſters were ordered every hour, and warm fomentations were unremittingly applied to the whole abdomen.

On viſiting her the next day, I found there had been no evacuation by ſtool: the pain had been more conſiderable, and every time the pills or any drink were given, the ſtomach rejected them almoſt immediately. The bleeding was repeated. Caſtor oil, whether given by itſelf, in gruel, or mixed with peppermint water, ſhared the ſame fate as every thing elſe;

I therefore ordered two or three grains of opium to be given immediately; and an hour after, various purgative medicines, and clyſters of infuſion of tobacco, were again to be perſevered in. The warm bath was ordered, and the fomentations were to be continued in the intervals.

This plan proved equally as unſucceſsful as the preceding, and I found my patient was evidently worſe.

I feel no heſitation in acknowledging I was entirely at a loſs with reſpect to the cauſe of this diſeaſe, and found no difficulty in perſuading myſelf it would remain inſcrutable, till ſuch time as the affair was cleared up by diſſection.

I had previouſly inquired whether there were any ſwellings about the belly, and was anſwered in the negative. I was in hopes there might be a collection of hardened fæces in the rectum; but, from the account of the nurſe who gave the clyſters, that could not be ſuppoſed to be the caſe. Being unwilling, however, to rely on the information of the nurſe, I determined to be ſatisfied of theſe two particulars; but while I went into an adjoining room, the patient had the ſenſation of ſomething burſting ſuddenly denly within her, and there immediately followed a very plentiful ſtool, mixed with matter, which procured her almoſt inſtant relief. Wiſhing to be ſatisfied from whence this matter proceeded, I deſired the women to ſearch; but they not agreeing in their report, I was deſired to examine, and perceived it to flow from the vagina. On introducing my finger, the diſcharge increaſed; but, on the ſtricteſt examination, I was not able to determine the preciſe part from whence it came. All circumſtances, however, being conſidered, I think there is greater probability it was ſeated in the cellular membrane, connecting the vagina and rectum, than in any other part. A clyſter was now injected, and it paſſed very freely. From this time ſtools were paſſed very regularly, and ſhe ſoon became perfectly well.

*Graveſend,*
April 17, 1787.

XII. *On*

